# Concordant Patterns of Population Genetic Structure and Symbiont Communities in a Broadcasting Spawning Coral Along a Western Australian Fringing Reef

**DOI:** 10.1002/ece3.72585

**Published:** 2026-01-28

**Authors:** Shannon L. Duffy, W. Jason Kennington, Zoe T. Richards, Luke Thomas

**Affiliations:** ^1^ School of Biological Sciences The University of Western Australia Crawley Western Australia Australia; ^2^ Australian Institute of Marine Science Perth Australia; ^3^ Centre for Evolutionary Biology, School of Biological Sciences The University of Western Australia Crawley Western Australia Australia; ^4^ Coral Conservation and Research Group, Trace and Environmental DNA Laboratory, School of Molecular Life Sciences Curtin University Bentley Western Australia Australia; ^5^ Collections and Research Western Australia Museum Welshpool Western Australia Australia

**Keywords:** *Acropora*, connectivity, genetic diversity, isolation by distance, population genomics, Symbiodiniaceae

## Abstract

The degree of connectivity across ecosystems is a key determinant of resilience, directly influencing recovery potential after disturbance and long‐term ecosystem stability. In reef‐building corals, there is added complexity to these processes because both the coral host and their symbiotic dinoflagellates determine resilience. Given these complexities, we investigated the connectivity of a broadcast spawning coral and its associated algal symbiont communities along the Ningaloo Reef Marine Park and Muiron Island Management Area. Using reduced representation sequencing and DNA metabarcoding in 158 colonies of *Acropora* cf. *tenuis* across 14 sampling sites, we detected significant spatial genetic structure in the coral host consistent with a pattern of isolation by distance (IBD). Spatial Autocorrelation analyses revealed that the genetic neighbourhood extends up to 50 km suggesting that this coral species has multiple demographically independent populations across Ningaloo Reef. Symbiont communities were dominated by *Cladocopium* and followed a similar IBD pattern of between‐site differences in community composition. We did not identify a significant correlation between host genetic diversity and symbiont community diversity at the colony level. However, spatial patterns of genetic differentiation between sample sites for the host and symbiont community composition were significantly associated suggesting that connectivity along a fringing reef system for both coral hosts and their symbionts is driven by similar biogeographic factors.

## Graphical abstract

We explored fine‐scale patterns of connectivity and symbiont associations across the Ningaloo reefscape to inform on post‐disturbance recovery, larval dispersal capabilities, and recruitment dynamics. We detected low but significant population genetic structure among sample sites spread across Ningaloo Reef with the highest diversity in southern sites. There is substantial evidence of isolation by distance, with an increasing signal of genetic differentiation with increasing geographic distance between sites. Interestingly, symbiont ITS2 metabarcoding revealed a positive association between coral genetic differentiation and symbiont community dissimilarity across sites, indicating that both are structured by shared biogeographic drivers.

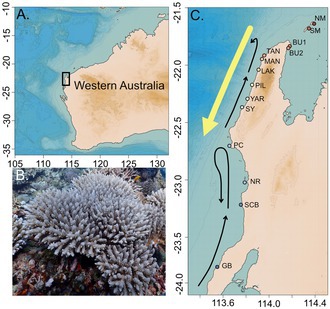

## 1 Introduction

Recovery following a disturbance event is an important component of resilience and is strongly driven by the connectivity of species within and between populations (Hock et al. 2017). This connectivity is facilitated through dispersal, and results in the exchange of individuals among populations promoting genetic diversity, enhancing adaptive potential, and ensuring the recolonisation of disturbed areas (Taylor et al. 1993). When barriers to dispersal are low, populations can more readily repopulate areas affected by disturbances, such as storms, wildfires, or human activities, thereby maintaining ecosystem functionality and stability (Bernhardt and Leslie 2013). On the other hand, when populations are isolated, the rate of recovery can be slow, demographic stochasticity can increase local extinction, and both species abundance and genetic diversity can decrease (Ceccarelli et al. 2011; Shoemaker et al. 2020).

Understanding and promoting connectivity is particularly important in the context of rapid environmental changes, where resilient ecosystems are better equipped to cope with changing conditions and sustain the ecological and economic services they provide (Chase et al. 2020; Gaitán‐Espitia and Hobday 2021). The ability of species to genetically adapt to new ocean warming pressures depends on the amount of genetic variation within the population on which natural selection can act (Barrett and Schluter 2008). As a result, genetic changes can occur under extreme temperature conditions, as evidenced by shifts in allelic variation at loci linked to thermal tolerance traits (Bay and Palumbi 2014; Fuller et al. 2020). Ideally, resilient corals that survive environmental stresses can disperse and contribute their adaptive traits to the broader population, enhancing overall population resilience (Nyström and Folke 2001).

Tropical reefs are often highly fragmented ecosystems, typically found fringing coastlines and islands (Kennedy and Woodroffe 2002), and marine organisms have evolved larval dispersal stages to navigate this landscape (Burgess et al. 2016; Chesson 1998). This dispersal period can vary but typically occurs across 1–2 weeks (Harrison and Wallace 1990), however, there is some evidence to suggest some species of coral have much longer larval stages (Graham et al. 2008; Randall et al. 2024). As a result, corals can disperse over distances of metres to 100 s of kilometres depending on species, oceanographic patterns or even storm activity (Bode et al. 2018; Connolly and Baird 2010; Prata et al. 2024; Radford et al. 2014; Torda et al. 2013; Underwood et al. 2020, 2009). Because dispersal is often constrained by distance, coral populations often reflect a stepping stone model of dispersal with strong patterns of isolation by distance (IBD) (Catalano et al. 2021).

The highly diverse family of dinoflagellate algae, *Symbiodiniaceae* (Davies et al. 2023; LaJeunesse et al. 2018), lives in symbiosis with corals and is significantly involved in the colonies' heat stress response (Berkelmans and van Oppen 2006; Matz 2024; Stat et al. 2006; van Oppen 2024). This resilience is often mediated by the presence of specific symbiont types, such as *Durusdinium trenchii* and *Symbiodinium microadriaticum* , which are known to confer enhanced thermal tolerance (Berkelmans and van Oppen 2006; Cantin et al. 2009; Lesser 2019; Swain et al. 2017). When the coral is under stress, the composition of their symbiont communities can shift, resulting in an increase in the relative abundance of those coined as more resilient, which can boost the coral holobiont's capacity to survive short‐term thermal stress (Bay et al. 2016; Grottoli et al. 2014). In contrast to brooding corals that predominantly acquire symbionts vertically (from the parent), most broadcast‐spawning corals acquire symbionts horizontally from the environment (Baird et al. 2009; Cumbo et al. 2013; Wilkinson and Sherratt 2001). This horizontal acquisition may confer greater flexibility, allowing coral recruits to acquire novel strains that are better suited to local environmental conditions, thereby enhancing their fitness (van Oppen et al. 2011). While the presence of “resilient” symbionts is important, colonies with diverse community compositions with the presence of more rare or background symbionts may also be important for maintaining functionality in a changing ocean (Coffroth et al. 2010; Gardner et al. 2019).

The intricate relationship between coral hosts and their symbionts complicates our understanding of resilience mechanisms within coral populations. Given the critical role this relationship plays in thermal tolerance and recovery, it is important to understand how these dynamics correspond with the host's connectivity and resilience. The few studies that have measured connectivity in both the coral host and its symbiont communities have largely focused on broad spatial scales, revealing concordant patterns of host and symbiont genetic variation structured by geography (Kenkel et al. 2013; Matias et al. 2023; Rose et al. 2021). However, little is known about how these patterns covary across finer spatial scales occurring within tens of kilometres, where local dispersal processes and microhabitat variation may influence host–symbiont associations. The aim of this study is to investigate the fine‐scale connectivity of a coral host and its symbiont community along the Ningaloo Reef Marine Park and the Muiron Islands Marine Management Area, located in Western Australia. Combined, this fringing reef spans over 300 km of coastline and 604,500 ha (Vanderklift et al. 2020). Reduced representation genomic sequencing was used to examine population structure in a widespread broadcast spawning *Acropora* coral and DNA metabarcoding of the ITS2 region to examine the *Symbiodiniaceae* diversity. Although connectivity in the symbionts was not measured directly, insight was gained by analysing between‐site differences in the symbiont communities associated with the coral host. Understanding these patterns, and levels of connectivity between adult populations, offers insight into the acclimation and recovery capacity of corals along Ningaloo Reef.

## 2 Methods

### 2.1 Sample Sites and Collection

**FIGURE 1:**
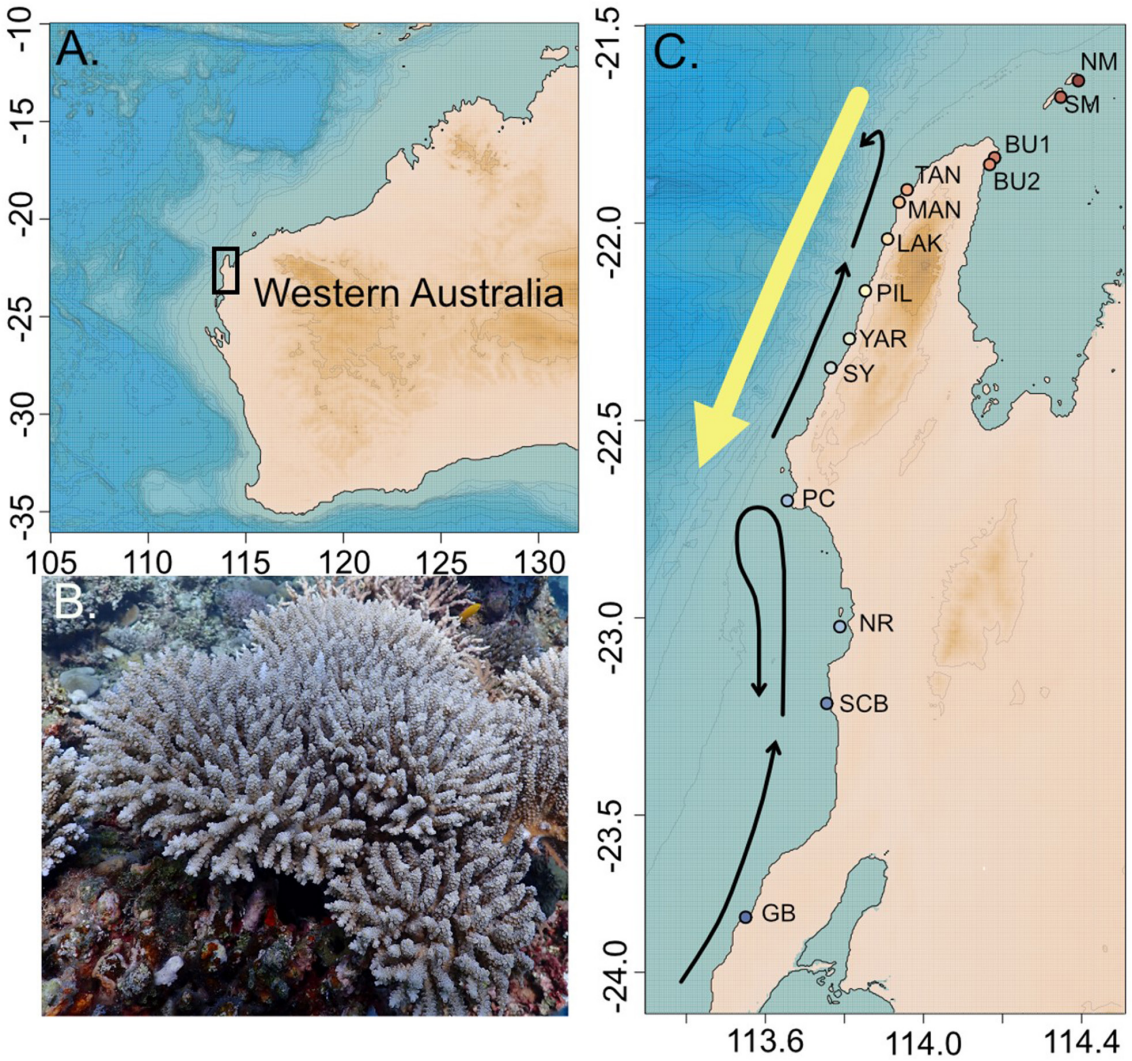
(A) Map of Western Australia showing the location of Ningaloo Reef; (B) Image of *Acropora* cf. *tenuis* collected from Ningaloo Reef for this study; (C) Map of Ningaloo Reef region and sites used for this study. Arrows represent major currents in the region, the yellow showing the Leeuwin Current and the black showing the Ningaloo Current (Vanderklift et al. 2020).

Sites were chosen to span the length of the Ningaloo Reef Marine Park and included the Muiron Islands Marine Management Area to the north (Table S1), from this point on referred to collectively as Ningaloo Reef. We categorised the sites into one of three regions based on oceanographic water currents along this reef system (Lowe et al. 2012; Woo et al. 2006). The Exmouth Gulf, encompassing the Muiron Islands Marine Management Area and Bundegi Reef (North and South), is dominated by strong localised tidal currents with upwelling near the northern tip of the cape (Verspecht 2002). On the west side, Point Cloates is a zone that disrupts the connectivity of Ningaloo, breaking it into a north and south (Woo et al. 2006). This is driven by the shape of the continental shelf, narrowing north of Point Cloates to < 10 km offshore, resulting in a disruption of the movement of the Ningaloo current moving towards the equator (Woo et al. 2006). Sites north of Point Cloates see more upwelling, eddies, and wave motion (Figure 1).

Tissue sub‐samples (approx. 2 cm) of *Acropora* cf. *tenuis* were collected from 20 georeferenced coral colonies along a 250 m transect at each site at least 2 m apart. Samples were identified in the field according to the morphological description provided by Wallace (1999) and confirmed with colony images taken in situ by WA Museum Coral Curator Z Richards. The open nomenclatural term cf. is used for our target species given the uncertainty that was raised about the identity of Western Australian *Acropora tenuis* in Bridge et al. (2024, albeit based on a single individual). Samples were flash frozen and stored at −80°C until they were sent to Diversity Array Technology Pty Ltd. (DArT P/L) using a reduced representation sequencing protocol where DNA extraction, library preparation, sequencing and SNP calling was undertaken. Similar to other RADseq protocols that generate genome‐wide single nucleotide polymorphism (SNP) data, DArTseq is a reduced representation sequencing library preparation method that uses specific site associated restriction enzymes. It is a widely used approach for exploring the genetic structure in species that lack sophisticated reference genome assemblies. DArT generated a genome‐wide SNP dataset using a Genotype by Sequencing approach (https://www.diversityarrays.com/) on the Illumina Hiseq2500. Sequences underwent primary bioinformatics at DArTseq where optimal SNPs were called (Kilian et al. 2012). The resulting reference sequences for all DArT loci were mapped against the *A. tenuis* reference genome (Cooke et al. 2020) to remove any possible symbiont contamination.

To identify the diversity of *Symbiodiniaceae* communities in these corals, a subset of 10 samples from each site was also sent to the Australian Genome Research Facility (AGRF) (https://www.agrf.org.au) to undergo DNA metabarcoding following in‐house DNA extraction using Qiagen DNA Mini Kit (Qiagen, Hilden, Germany) following the manufacturer's procedure. The Internal Transcribed Spacer 2 (ITS2) region of the *Symbiodiniaceae* ribosomal DNA was amplified using polymerase chain reaction (PCR). This was done using symbiont‐specific forward and reverse primer pair: ITSD (5′‐GTGAATTGCAGAACTCCGTG‐′3) and ITS2‐rev2 (5′‐CCTCCGCTTACTTATATGCTT‐′3) (Stat et al. 2009). Amplicons underwent library preparation with secondary PCR to attach flow cell adapters and unique barcodes. Then pooled at equal molar concentrations and sequenced on Illumina MiSeq using the V2 500 cycle paired‐end kit. Sequenced reads were returned as de‐multiplexed fastq.gz output files with barcodes and adapters (non‐biological nucleotides) already removed.

### 2.2 Filtering and Quality Control

The genome‐wide Single Nucleotide Polymorphism (SNP) dataset generated by DarT (https://www.diversityarrays.com/) was filtered using the *DartR* package (Gruber et al. 2018) resulting in a genotype matrix for downstream analysis (Table S2). First, secondary SNPs were removed which ensures that quality is prioritised before removing SNPs based on their proximity to each other. Next, loci were filtered based on read depth, with loci having a mean read depth below 5 and above 200 being excluded to ensure sufficient coverage for confident heterozygosity calls while avoiding the inclusion of paralogs. Loci reproducibility was then assessed, retaining those with reproducibility scores above 0.95 to ensure reliable base calls, as low reproducibility indicates unreliable loci. Subsequently, loci with a call rate below 0.7 were removed to eliminate SNPs with excessive missing data, reducing noise and computational load. Following this, individuals with a call rate below 0.75 were excluded to remove outliers that might skew the dataset due to poor sequencing performance. Monomorphic loci were then filtered out as they no longer provide variability post‐individual removal. Finally, loci with minor allele frequencies below 0.02 were removed to discard rare alleles.

We sequenced multiple individuals twice to establish a threshold for removing clones that could be possible from sampling due to vegetative fragmentation; this also gave a baseline differentiation to identify any clones we may have potentially sampled. Prior to carrying out any population‐level analyses, an initial screening to identify any cryptic species or clones in the filtered dataset was done using principal components analyses (PCA) and visual inspection of dendrogram and neighbour‐joining tree created with raw read data (Figure S1) (Matz 2018). All samples that appeared as clones and outliers were removed from downstream analyses.

The SNP dataset was run through *PCadapt* (Privé et al. 2020), *Bayescan* (Foll and Gaggiotti 2008), and *OUTflank* (Whitlock and Lotterhos 2015) to identify loci influenced by selection. Those present in two of the three analyses would be removed in accordance with the assumption of neutrality (Gautier 2015) and to account for the potential influence of false positives known to be an issue with current outlier detection methods (Hoban et al. 2016; Lotterhos and Whitlock 2014). No outlier loci were identified with *OUTflank*, 49 loci identified as outliers using *Bayescan*, and 26 loci with *PCadapt*. However, since there were no overlapping loci among methods and one detected no outlier loci, none were removed from the SNP dataset.

### 2.3 Genetic Diversity and Population Structure

To measure the genetic diversity, gene diversity (*H*_E_), observed heterozygosity (*H*_o_), and allelic richness (*A*_R_) were calculated for each site using the *hierfstat* package (Goudet 2005). To test for statistical differences between sites we used the Friedman test and pairwise Wilcoxon tests in the *stats* package (R Core Team 2021). We explored patterns of spatial genetic structure using a combination of approaches. Pairwise *F*_ST_ was estimated between sites using *StAMPP* (Pembleton et al. 2013). To determine the number of genetic populations present without any prior information on the geographic data, we performed a Bayesian clustering analysis using *Structure* (Porras‐Hurtado et al. 2013) measuring for up to 10 possible genetic clusters (*K*) running 10 iterations of 100,000 repetitions (burning 10,000) for each value of *K*. Variations of *K* were summarised using *Clumpak (* https://tau.evolseq.net/clumpak/) and then plotted in R, with optimal *K* determined by examining the ln Pr(*X*|*K*) and delta *K* plots (Evanno et al. 2005). Population genetic structure was also assessed using a discriminant analysis of principal components (DAPC) plot to observe the genetic distance between each population without the strict assumptions of *STRUCTURE* (Jombart and Collins 2015). The DAPC was created using the *adegenet* package, retaining the first 13 principal components (PCs) based on the *k* − 1 criterion, where *k* = 14 is the number of populations defined based on sampling locations (Thia 2022). The DAPC analysis was performed with two discriminant functions to maximise the genetic differentiation among the sites and visualise structure.

To test for IBD, we compared matrices of genetic differentiation (pairwise *F*_ST_) and geographic distance using a Mantel test in the R package *vegan* (Oksanen et al. 2024). The geographic distances were based on the over water distance (OWD), which accounted for land masses and ocean bathymetry up to 100 m deep between coordinates, calculated using the R package *marmaps* (Pante and Simon‐Bouhet 2013). To further explore the patterns of dispersal across the reefscape, we used a hierarchical Analysis of MOlecular VAriance (AMOVA) (Excoffier et al. 1992) to partition genetic variance between regions (Gulf, North and South), between sites within regions and within sites between individuals with the *poppr* package (Kamvar et al. 2014). While this method can estimate variance at an individual level we took a conservative approach in the AMOVA and didn't partition within individuals as the assumption of Hardy–Weinberg Equilibrium may not be met. To test the significance of variations *randtest* from *ade4* was used on the AMOVA output (Dray and Dufour 2007). Finally, to determine the genetic neighbourhood and the extent of dispersal, we used *GenAlex* v 6.503 (Peakall and Smouse 2006, 2012) to calculate the spatial autocorrelation coefficient (*r*) over a range of OWD classes. Positive *r* values indicate genetic similarity between individuals and the distance class where *r* no longer differs significantly from zero provides an approximation of the genetic neighbourhood, the distance over which random mating occurs (Peakall et al. 2003). The *r* values were plotted against distance class to produce a spatial genetic autocorrelogram and tests for statistical significance were determined using random permutations (999) and calculating the 95% confidence limits by bootstrapping (Peakall et al. 2003).

### 2.4 Symbiodiniaceae Diversity

Demultiplexed fastq files were processed using the SymPortal analytical framework (https://symportal.org) (Hume et al. 2019). The count matrix output displays the number of reads for defining intragenomic variants (DIVs) identified within each sample, discarding any types that produce < 200 sequence reads. DIVs are recurring ITS2 sequences that can reflect both intra‐ and intergenomic variation within *Symbiodiniaceae* lineages, meaning that some of the observed diversity arises from multiple sequence variants within a single symbiont genotype rather than from distinct species (Hume et al. 2019). In an attempt to account for this SymPortal also determines ITS2 type profiles for each individual colony, these profiles collate specific sets of recurring DIVs that represent distinct *Symbiodiniaceae* taxa genotypes defined by the most dominant or co‐dominant DIVs. As a result, while we use DIVs to calculate diversity metrics and test *Symbiodiniaceae* community composition between sites and individuals we interpret this variant diversity with caution, using the ITS2 profiles instead to define the dominant *Symbiodiniaceae* taxa present at Ningaloo. From the DIV count matrix, alpha diversity was quantified by calculating Shannon's diversity index (measure of richness and evenness), and DIV richness (number of DIVs per colony) using the *vegan* package in R. Prior to all calculations the relative abundance of DIVs were square root transformed to reduce the weighting of abundant variants and ensure all background variants would be captured in the diversity.

Seasonal shifts in symbiont communities have been observed independently of bleaching (Carballo‐Bolaños et al. 2019; Chei et al. 2025; Chen et al. 2005; Ziegler et al. 2015), such as the summertime increase in the relative abundance of *Durusdinium* in *Leptoria phrygia* in Taiwan (Huang et al. 2020). Since our samples were collected over various time points and across multiple seasons, we included collection season/year as a random effect in all models. To test for significant differences in diversity (Shannon's and DIV richness) across sites, we ran generalised linear mixed models with *glmmTMB* (Brooks et al. 2017) in R with Gaussian family distribution, site as the fixed effect and collection season included as a random effect. Residual diagnostics indicated that model assumptions were met and AIC comparisons with equivalent linear mixed models showed that the *glmmTMB* provided a better fit. *Symbiodiniaceae* population diversity (differences in DIV composition between individual colonies) was visualised using a DAPC plot in *adegenet* (Jombart 2008) and a Permutational Multivariate Analysis of Variance (PERMANOVA) was used to test for significant differences between sites using the *adonis2* function in *vegan*. We also performed Mantel tests to test for IBD in symbiont communities across sites, using matrices of pairwise Bray–Curtis distances (calculated using a pooled DIV count matrix) and pairwise geographic distances based on the OWD between sites.

### 2.5 Correlations Between the Coral Host and Symbiont

To examine whether corals with higher genetic diversity host more diverse symbiont communities we performed linear mixed effects models (LMM) with the *lme4* package (Bates et al. 2015). With these models host coral observed heterozygosity was used to predict the individual colony Shannon's diversity estimates based on their DIV profiles. We accounted for non‐independence among samples by including site as a random effect. To test whether genetically similar corals share similar symbiont communities, we conducted Mantel tests using pairwise host genetic distances based on allele frequency shifts and pairwise Bray–Curtis distances of DIV abundances. Both individual‐level analyses were run on a subset of the colonies where both host and symbiont data were available (*n* = 74). To explore how spatial genetic structure in the host and *Symbiodiniaceae* community composition co‐varied across the reef, we also performed an additional site‐level Mantel test using pairwise *F*_ST_ (coral host) and pairwise Bray–Curtis distances calculated from pooled DIV abundances within each site (symbiont). The Mantel tests were run in *vegan* with 999 permutations.

## 3 Results

### 3.1 Genetic Diversity in the Coral Host

DArT genotyping returned 26,393 loci called across 185 individual colonies representing 14 sites. After filtering and the removal of 3 clones (for information on clone classification refer to Methods S1), 5368 loci remained across 158 individuals (Table 1). Estimates of genetic diversity showed relatively stable patterns across our sample sites (Table 1). While the Friedman rank sum test indicated significant differences among sites (gene diversity *p* < 0.001; allelic richness *p* < 0.001), subsequent pairwise Wilcoxon tests adjusted for false discovery rates (FDR) indicated there were significant differences in only a few between‐site comparisons (Table S3).

**TABLE 1:** Information of sampling sites, host coral diversity based on 5368 loci, and symbiont diversity metrics. Number of samples (*N*), number of samples post quality control filtering (*NPQC*), gene diversity (*H*_E_), observed heterozygosity (*H*_O_), and allelic richness (*A*_R_), and symbiont number of samples (*N*), Shannon diversity index (*H*) and DIV Richness (S).

Site	Site	Host					Symbiont		
	Code	*N*	NPQC	*H*_E_	*H*_O_	*A*_R_	*N*	*H*	*S*
North Muiron	NM	14	12	0.243 ± 0.002	0.149 ± 0.002	1.238 ± 0.002	10	1.88 ± 0.06	8.7 ± 0.63
South Muiron	SM	20	15	0.246 ± 0.002	0.156 ± 0.002	1.242 ± 0.002	10	2.10 ± 0.11	11.5 ± 1.60
North Bundegi	BU1	15	13	0.243 ± 0.002	0.150 ± 0.002	1.239 ± 0.002	10	1.96 ± 0.05	9.4 ± 0.48
South Bundegi	BU2	20	12	0.244 ± 0.002	0.153 ± 0.002	1.239 ± 0.002	10	2.67 ± 0.21	21.5 ± 3.83
Tantabiddi	TAN	13	6	0.245 ± 0.003	0.156 ± 0.002	1.235 ± 0.003	10	1.97 ± 0.07	9.6 ± 0.69
Mangrove	MAN	8	8	0.243 ± 0.003	0.161 ± 0.002	1.236 ± 0.003	10	2.43 ± 0.13	16.5 ± 2.20
Lakeside	LAK	12	11	0.241 ± 0.002	0.153 ± 0.002	1.236 ± 0.002	10	2.81 ± 0.21	25.1 ± 4.18
Pilgonaman	PIL	13	11	0.246 ± 0.002	0.156 ± 0.002	1.241 ± 0.002	10	2.98 ± 0.20	28.8 ± 4.23
Yardie Creek	YAR	8	6	0.24 ± 0.003	0.156 ± 0.002	1.231 ± 0.003	10	2.82 ± 0.26	26.8 ± 5.32
South Yardie	SY	16	12	0.245 ± 0.002	0.162 ± 0.002	1.241 ± 0.002	10	2.78 ± 0.24	25.2 ± 4.81
Point Cloates	PC	18	11	0.241 ± 0.002	0.159 ± 0.002	1.236 ± 0.002	10	3.34 ± 0.10	35.3 ± 2.59
North Reef	NR	16	13	0.239 ± 0.002	0.159 ± 0.002	1.236 ± 0.002	10	2.50 ± 0.28	20.5 ± 4.70
South Coral Bay	SCB	18	12	0.238 ± 0.002	0.155 ± 0.002	1.234 ± 0.002	10	2.91 ± 0.21	26.9 ± 4.31
Gnaraloo Bay	GB	19	16	0.237 ± 0.002	0.163 ± 0.002	1.234 ± 0.002	10	3.07 ± 0.20	30 ± 4.12

### 3.2 Spatial Genetic Structure in the Coral Host

**FIGURE 2:**
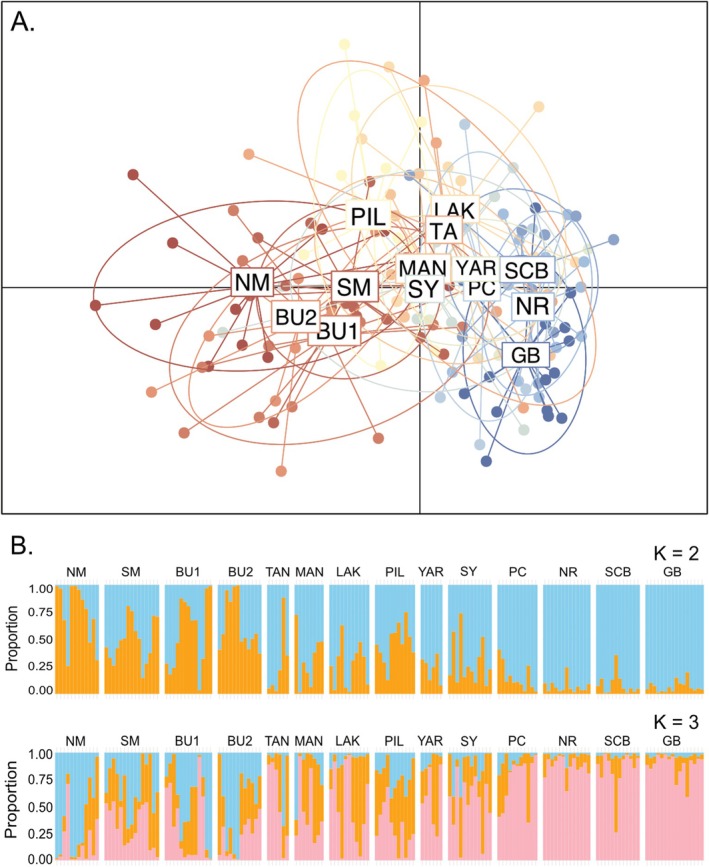
(A) Discriminant analysis of principal components (DAPC) based on 13 Principal Components and 2 discriminant functions retained. Ellipses represent 95% confidence inertia ellipses and dots represent individuals. (B) Assignment probabilities based on the Bayesian clustering analysis where each bar represents an individual and sites are ordered by latitude, *K* = 2 was best fit based on Evanno method (Evanno et al. 2005), with *K* = 3 included for comparison. Sampling sites are ordered by increasing latitude.

The Bayesian clustering analysis found evidence of *K* = 2 as the most likely number of populations based on the Evanno method (Figure S2). Samples collected from southern sites showed higher membership to cluster 1 and those from sites north of Point Cloates showed higher membership to cluster 2, but there was evidence of admixture between the two clusters across all sites (Figure 2B). The DAPC showed a general pattern of genetic dissimilarity increasing with distance, with some separation between the gulf, northern, and southern regions (Figure 2A). This general transition from north to south was also evident in the scatterplot based on PCA (Figure S1). In support of these broad spatial patterns, global estimates of genetic differentiation (*F*_ST_) showed low, but significant spatial genetic structure across our study sites (*F*_ST_ = 0.0047, *p* = 0.005). The AMOVA showed there was low, but significant variation among regions, but not among sites within regions (Table 2). Pairwise *F*_ST_ values among sample sites generally supported this pattern, with low or non‐significant *F*_ST_ values occurring between geographically proximate locations, and higher and significant values between geographically more distant sites. However, significant divergences were detected between sites within the same region (Table S4).

**TABLE 2:** The analysis of molecular variance (AMOVA) for coral host genetic variation and PERMANOVA for symbiont community composition, reporting the proportion of total variation explained at each level and the corresponding significance level. Significant *p*‐values are in bold.

	Coral host		Symbiont	
	% total variance	*p*	% total variance	*p*
Within site (between samples)	98.9	**0.001**	66.2	
Within region (between sites)	0.18	0.074	17.72	**< 0.001**
Between regions	0.92	**0.001**	16.07	**< 0.001**

**FIGURE 3:**
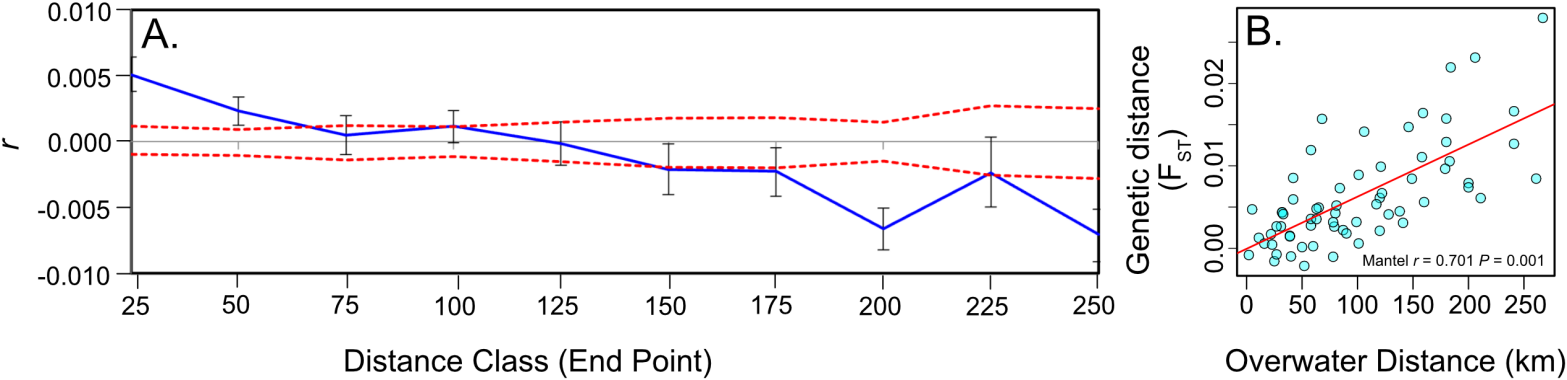
(A) Spatial autocorrelogram plot based on all individuals based on site over water distances (km) with 95% confidence intervals presented, *r* is the blue line and U and L are the red dashed line. (B) Relationship between pairwise genetic distance (*F*_ST_) and over water distance (km).

This pattern was confirmed with a Mantel test that showed a strong pattern of IBD (*r* = 0.71, *p* = 0.001) (Figure 3B). There was also evidence of IBD in the northern sites only (*r* = 0.42, *p* = 0.035), but not in the southern sites (*r* = 0.17, *p* = 0.375). Spatial autocorrelation analysis revealed significant positive *r* values in the first two distance classes before *r* became non‐significantly different from 0 within the 51–75 km distance class with the *x* intercept in the spatial autocorrelogram occurring at 122.3 km (Figure 3A).

### 3.3 Symbiont Community Diversity

**FIGURE 4:**
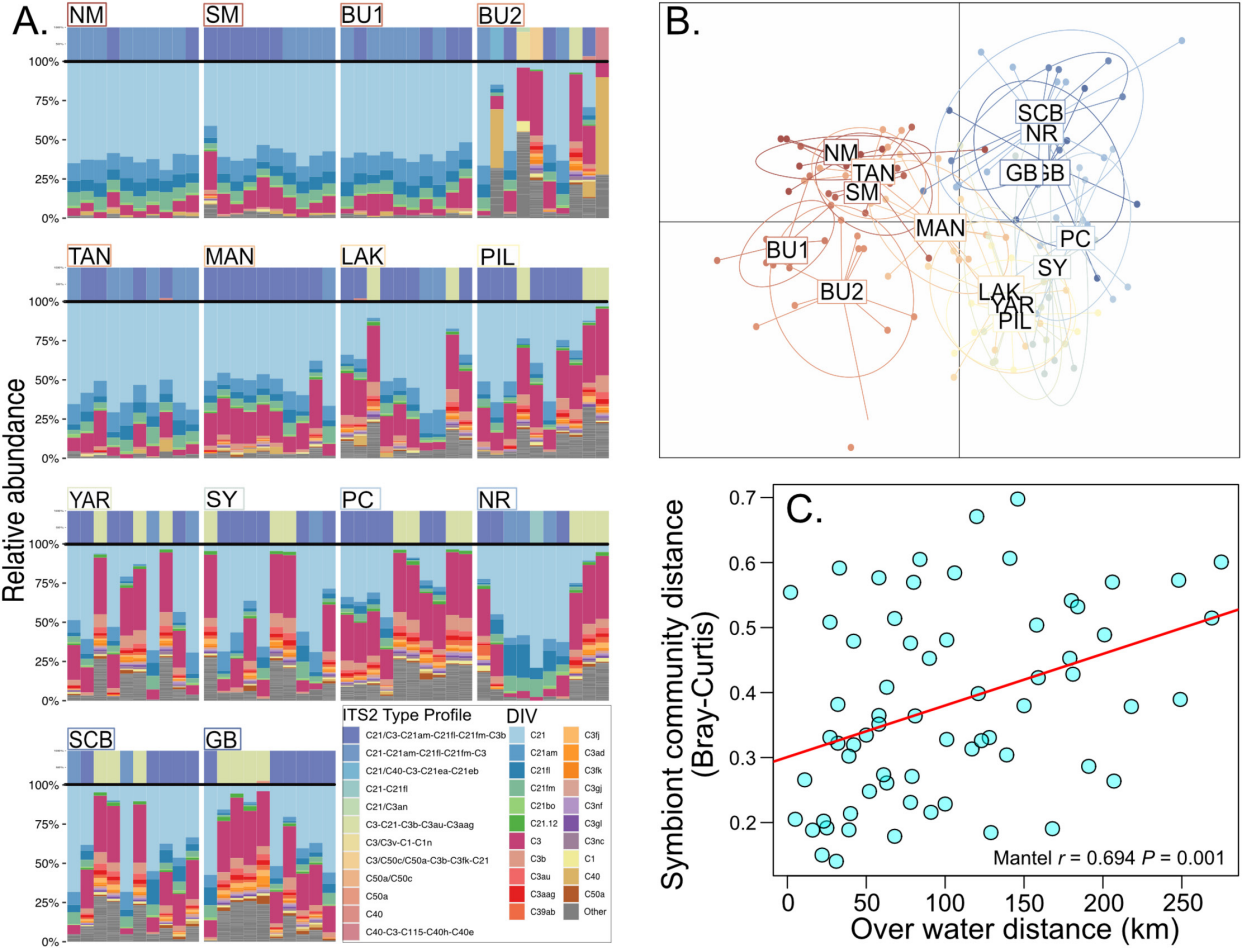
*Symbiodiniaceae* community composition and sequence diversity along the Ningaloo Reef Marine Park. Sites are ordered based on latitude, with warm colours denoting northern sample sites and cooler colours for southern sites. (A) Stacked bar plots of relative *Symbiodiniaceae* ITS2 DIVs abundances in sites samples along Ningaloo Reef Marine Park. Each column represents an individual that was sampled. The top colour represents the ITS2 profile type, and the colours below the black line represents the proportion of the DIV type present in that colony. (B) Discriminant analysis of principal components (DAPC) based on 13 Principal Components and 2 discriminant functions retained based on normalised DIV counts. Ellipses represent 95% confidence inertia ellipses and dots represent individuals. (C) Relationship between symbiont community composition distance (Bray–Curtis) and over water distance, red line represents positive linear relationship.

A total of 6,453,635 paired‐end reads were produced from the 150 individual colonies. Symbiont sequences were clustered into 194 unique DIVs all from the *Cladocopium* genus. SymPortal identified 12 ITS2 profiles with the number of DIVs per individual ranging from 4 to 49 (Figure 4A). The dominant ITS2 type profile (C21/C3‐C21am‐C21fl‐C21fm‐C3b) represented 51.3% of samples, with the second (C21‐C21am‐C21fl‐C21fm‐C3) at 28% and (C3‐C21‐C3b‐C3au‐C3aag) at 17.3%, with the remaining 3.3% comprising 7 minor profiles. Of these profiles C21 and C3 (*Cladocopium sodalum*) represented the most common symbiont at Ningaloo Reef.

All measures of alpha diversity revealed significant variation in symbiont diversity across sites (Shannon's index *p* < 0.001, DIV richness *p* < 0.001) (Figure S3), with models including site as a fixed effect explaining up to 40% of the total variance (Shannon's index *R* ^2^ = 0.389, DIV richness *R* ^2^ = 0.311). Point Cloates had the highest diversity, with Pilgonaman Bay and Gnaraloo Bay also having highly diverse communities. The lowest symbiont diversity was found in the northernmost sites such as North Muiron Island, Bundegi, and Tantabiddi (Table S5).

The DAPC based on a normalised DIV count matrix showed clear differences in Symbiodiniaceae population diversity between sites as well as separation of the northern to southern sites (Figure 4B). The PERMANOVA supported this, showing a significant between region and between site within region differences in symbiont community composition (Table 2). Additionally, a Mantel test showed that between site distance (Bray‐Curtis) was significantly associated with OWD (*r* = 0.395, *p* = 0.022; Figure 4C). This relationship was also evident in an analysis based on the northern sites only (*r* = 0.53, *p* = 0.015), but not the southern sites (*r* = −0.14, *p* = 0.500).

### 3.4 Host—Symbiont Correlations

**FIGURE 5:**
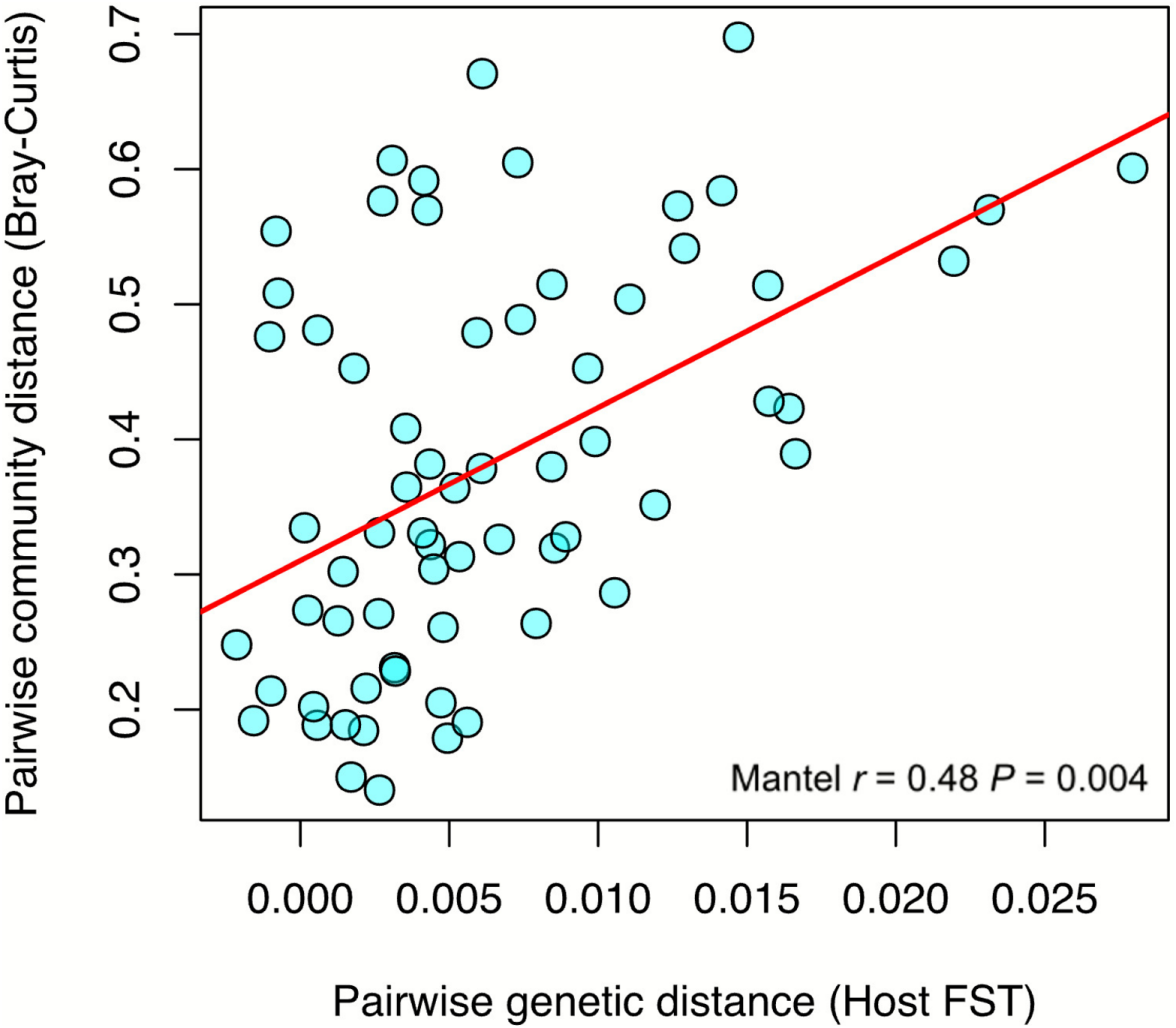
Scatterplot showing the relationship between pairwise genetic differentiation in the coral host and symbiont community composition distance. Each point represents a site pairwise comparison, and the red line represents the significant positive linear relationship between the two.

At the individual level, the mixed‐effects models revealed no significant relationship between individual host heterozygosity and symbiont diversity (richness: *p* = 0.906; Shannon: *p* = 0.754) after accounting for site‐level variation (Figure S4). Approximately 40% of variation in symbiont richness occurred among sites, indicating that local environmental or geographic factors, rather than host genetic diversity, predominantly shape symbiont community structure. Similarly, Mantel tests using pairwise host genetic distances and symbiont community dissimilarities across the same 74 shared colonies found no significant correlation (Mantel's *r* = −0.045, *p* = 0.799), suggesting that genetically similar hosts do not harbour more compositionally similar DIV assemblages. At the site level, there was a significant positive relationship between pairwise genetic divergences in the coral host (*F*_ST_) and the between‐site symbiont community differences (Bray‐Curtis distance) (Mantel's *r* = 0.481, *p* = 0.004), demonstrating concordant spatial structuring between host genetic differentiation and symbiont community across the reef (Figure 5). This relationship was also evident when including the northern sites only (*r* = 0.337, *p* = 0.047), but not when the southern sites only were considered (*r* = −0.531, *p* = 0.917).

## 4 Discussion

This study explored patterns of genetic connectivity and symbiont associations in a widespread broadcast spawning coral along a 300 km fringing reef in Western Australia. Our study revealed significant population structure across the study region, with evidence of regional differences coinciding with a geographical feature and patterns of IBD. Spatial autocorrelation revealed that positive genetic structure extended at least to 50 km suggesting large genetic neighbourhoods in this species of coral, with evidence of some dispersal up to 120 km. Symbiont communities were dominated by the genus *Cladocopium*. Despite this dominance, symbiont community diversity was high and varied significantly across sites, with the more diverse range of DIVs being harboured in the southern regions. There were no significant individual level host‐symbiont associations within reefs. However, at the site level, host genetic differentiation was positively correlated with differences in the symbiont community composition, suggesting that host‐symbiont associations are influenced by shared geographic structuring rather than direct genotype‐specific partnerships.

The *Acropora* species on which this study was based forms a cryptic species complex in Western Australia with two lineages based on reproductive timing (Gilmour et al. 2016; Rosser et al. 2020; Thomas et al. 2024, 2022). Despite this, our analysis found no evidence of multiple cryptic species existing along Ningaloo Reef, aligning with the previous suggestion that one of the two lineages in this species complex becomes less frequent at higher latitudes and is potentially absent from Ningaloo Reef (Rosser et al. 2020). While our genetic data provide no evidence of multiple cryptic species existing within the study area, there was evidence of low levels of population structure with two distinct populations, represented by sites situated north or south of Point Cloates. The boundary between these two groups coincides with a narrowing of the continental shelf north of Point Cloates (Woo et al. 2006). At this point in the reef the northward‐flowing Ningaloo Current (Taylor and Pearce 1999) interacts with the strong Leeuwin current that flows south along the continental shelf edge (Cresswell and Golding 1980). This interaction causes counter‐clockwise eddies to form around Point Cloates creating unique conditions that seem to generate a barrier to gene flow (Woo et al. 2006). Genetic divergences between sites north and south of Point Cloates have also been observed in other Ningaloo Reef taxa including the coral *Pocillopora damicornis* (Thomas, Kendrick, Stat, et al. 2014), the fish *Pomacentrus moluccensis* (Wilson et al. 2016), and *Sargassum* spp. (Fulton et al. 2014). We also found symbiont community composition varied significantly between sites situated either side of this landmark, further supporting the notion that the Point Cloates area acts as a biogeographic divide.

In addition to the genetic structuring between northern and southern sites, we also found significant genetic differentiation among sample sites, particularly in the north. Sampling across 300+ km of fringing reef revealed that 57% of the pairwise *F*_ST_ comparisons were significant (FDR adjusted) and 26% of symbiont diversity pairwise comparisons showed significant differentiation. The patterns of IBD found in both the coral host and symbionts across all sites, and across the northern sites only, suggest that dispersal follows a stepping‐stone pattern (Wright 1943) commonly observed in genetic studies on corals (Davies et al. 2015; Dennis et al. 2024; Evans et al. 2019; Prata et al. 2024; Smith et al. 2017). Although recent genetic studies on the same species have found no population structure within offshore reef systems in Western Australia (Thomas et al. 2024, 2022), this may reflect the smaller spatial scales over which sampling was conducted in these studies. Furthermore, the complex bathymetry and current patterns along Ningaloo Reef (Cassata and Collins 2008; Pomeroy et al. 2021; Taebi et al. 2011) may have restricted larval dispersal and lead to a gradual genetic divergence developing between sites. This could be what is occurring with sites in the Exmouth Gulf that consistently clustered apart from other northern sites. The combination of the biogeographic barrier imposed by the cape and the influence of the gulf's strong, localised tidal currents could be restricting the larval exchange between these two regions (Verspecht 2002; Grimaldi et al., pers. comm.). Interestingly, while broadcast spawners like *Acropora* typically show higher gene flow compared to brooding species who tend to exhibit more genetic structuring due to shorter dispersal periods (Baird et al. 2009; Thomas et al. 2020; Underwood et al. 2009), genetic subdivision is still evident along Ningaloo Reef in other broadcast‐spawning corals further supporting that biogeographic factors may be restricting connectivity along this reef (Evans et al. 2019; Whitaker 2004).

The strong positive relationship between genetic distances in the coral host and their symbiont community composition suggests that both are influenced by similar environmental or biogeographic factors driving between‐site differentiation. Importantly, the absence of outlier loci suggests the genetic patterns observed in the coral host are selectively neutral, arguing against local adaptation as the primary explanation. This is noteworthy given that evidence for local adaptation is commonly detected in coral studies, particularly over large geographical distances (Dixon et al. 2015; Fuller et al. 2020; Kenkel et al. 2013; Matz et al. 2018; Thomas et al. 2017) or in the presence of strong environmental gradients (Marhoefer et al. 2021; Thomas et al. 2022; Wang et al. 2019) and shifts in symbiont community composition have been linked with environmental factors over broader spatial scales (Baums et al. 2014, 2010; van Oppen et al. 2018). However, it is possible that adaptive variation was not captured by our reduced‐representation dataset, which samples only a proportion of the genome. Local adaptation may exist at loci not genotyped here or may be polygenic in nature, requiring higher‐resolution whole‐genome sequencing to detect. The general consistency in genetic diversity in the coral host across sites suggests that historical factors like population crashes are unlikely drivers of the current patterns. However, there remains the possibility that the association stems from a combination of IBD patterns in the coral host and environmental variation shaping between‐site variation in the symbionts.

The predominant *Symbiodiniaceae* taxa across all sites were C21 and C3 identified in the type profiles, both commonly found in *Acropora* species (Berkelmans and van Oppen 2006; Butler et al. 2023; Lewis et al. 2024; Silverstein et al. 2011; Thomas, Kendrick, Kennington, et al. 2014). Both these have been described as generalist symbionts (Butler et al. 2023). In the GBR, *Cladocopium* C21 has been suggested to favour inshore conditions (Tonk et al. 2013), where corals have been observed to suffer less severe bleaching, C3 has also been found inshore in the Kimberly's in Western Australia (Jung et al. 2021), and both have also been found in corals of the Red Sea (Santoro et al. 2025). However, it remains unclear whether these types constitute a resilient symbiont (Bay et al. 2016; Jones 2008) or a generalist (Lewis et al. 2024). Interestingly, two corals from South Bundegi were dominated by *Cladocopium madreporum* (C40), which is the dominant type for this *Acropora* spp. in Western Australia offshore reefs (Thomas et al. 2022). This site is heavily degraded and rubble‐dominated compared to the neighbouring Bundegi site to the north; however, it had the most diverse symbiont communities in the region. There is increasing literature showing rubble habitats contribute to an increase in the diversity of protist and microbiome communities (Borbee et al. 2023; Granados‐Cifuentes et al. 2015; Wolfe et al. 2021), and may explain why the rubble‐dominated benthic habitat had unexpectedly high symbiont diversity. It could also be a reflection of this community being under successional flux which may be leading to higher diversity whilst the community adjusts to a new stable state (Jentsch and White 2019).

The broadscale connectivity studies of corals suggest Ningaloo Reef is relatively isolated from other nearby reefs such as the Pilbara (Adam et al. 2022; Underwood 2009). There is evidence to suggest that Ningaloo is seeded by reefs to the north (Feng et al. 2015), but this connectivity is restricted to Exmouth Gulf and Dampier (Evans et al. 2021). Consequently, Ningaloo Reef is relatively isolated and reliant on self‐seeding following recovery, which can be slow as seen at Bundegi (Babcock et al. 2021), north of Point Cloates (Holmes et al. 2017), and in Coral Bay following spawning anoxia events (Newnham et al. 2020; Richards et al. 2024; Shedrawi et al. 2017). We found beyond 50 km, spatial autocorrelation diminished along the Ningaloo Reef system, suggesting limited gene flow over extended latitudinal distances. This dispersal information complements the current design of marine reserves along the Ningaloo Reef system. The presence of regularly spaced reserves or closures every 50–100 km will capture the reef's high genetic and symbiont community diversity and ensure proximity necessary for recovery after disturbances. Integrating genetic metrics with oceanographic modelling and larval particle tracking will refine our predictions of coral connectivity and would allow for further validating both methods (Burt et al. 2024; Galindo et al. 2006; Krueck et al. 2020; Matz et al. 2018). This integrated approach is particularly vital as rising temperatures might modify current regional circulation patterns (Boschetti et al. 2020; Fultona et al. 2011; Taebi et al. 2011), which could affect gene flow and population structure, making it imperative to refine predictive models under climate change scenarios.

## Author Contributions

**Shannon L. Duffy:** conceptualization (lead), data curation (lead), formal analysis (lead), funding acquisition (equal), investigation (equal), methodology (equal), project administration (lead), writing – original draft (lead), writing – review and editing (equal). **W. Jason Kennington:** conceptualization (equal), formal analysis (equal), methodology (equal), supervision (equal), writing – review and editing (equal). **Zoe T. Richards:** data curation (equal), writing – review and editing (equal). **Luke Thomas:** conceptualization (equal), data curation (equal), funding acquisition (equal), methodology (equal), resources (equal), supervision (equal), writing – review and editing (equal).

## Funding

This research was conducted under the AIMS@UWA alliance. This work was supported by the University of Western Australia, Australian Institute of Marine Science, and the Minderoo Foundation. It was funded by the Royal Society of Western Australia through the John Glover Research Grant, the Minderoo Foundation, and the Ecological Society of Australia's Holsworth Research Grant.

## Conflicts of Interest

The authors declare no conflicts of interest.

## Supporting information


**Appendix S1:** ece372585‐sup‐0001‐AppendixS1.xlsx.

**Appendix S2:** ece372585‐sup‐0002‐AppendixS2.docx.

## Data Availability

The datasets generated and analysed for this research are available with the R script on the Open Science Framework https://osf.io/8ae5u/.
