# Supplementary material for: Concordant Patterns of Population Genetic Structure and Symbiont Communities in a Broadcasting Spawning Coral Along a Western Australian Fringing Reef
Source: Ecol Evol. 2026 Jan 28;16(1):e72585. doi: 10.1002/ece3.72585 (PMC12848603; doi:10.1002/ece3.72585)
Supplement: Supplementary file 2 — Appendix S2: ece372585‐sup‐0002‐AppendixS2.docx. [file ECE3-16-e72585-s001.docx]

Concordant patterns of population genetic structure and symbiont communities in a broadcasting spawning coral along a Western Australian fringing reef

Supplementary Figures


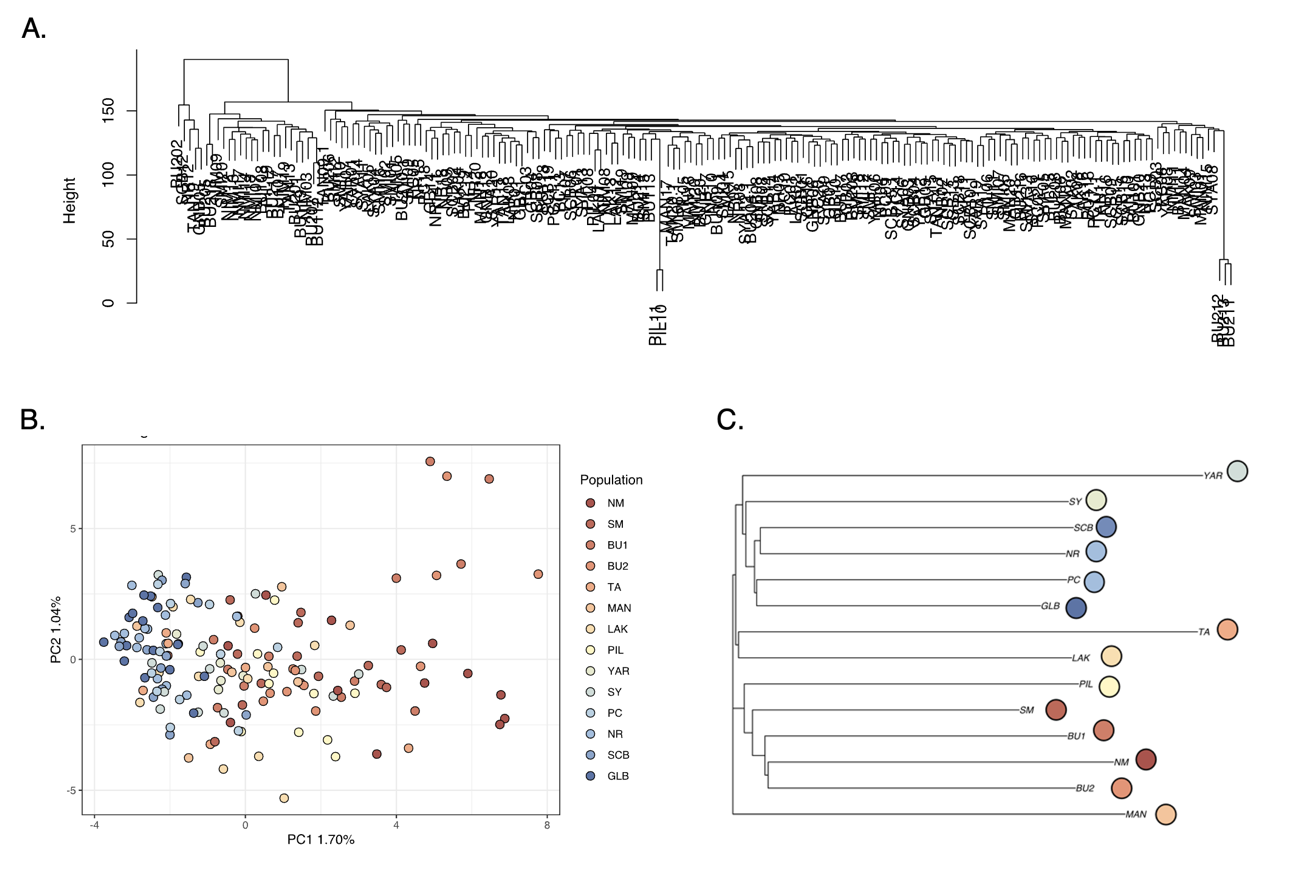


Supplementary Figure 1: Visual assessment of Single Nucleotide Polymorphism (SNP) data prior to filtering to inspect for clones and outliers. A) Dendrogram of genelight data matrix with clones included. B) Principal Component Analysis plot after clones were removed.

C) Neighbour-joining tree summarising genetic similarity of sites based on Euclidean distances.


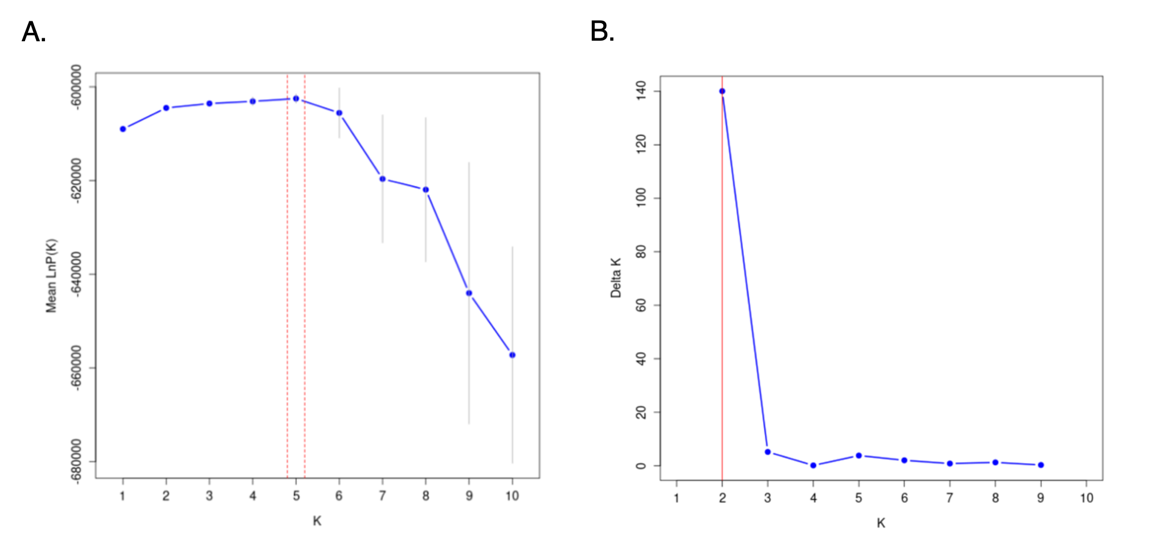


Supplementary Figure 2: Mean estimates of the A) log probability and B) delta K for each value of K arising from the STRUCTURE analysis. Error bars for log probability estimates are in standard deviations.


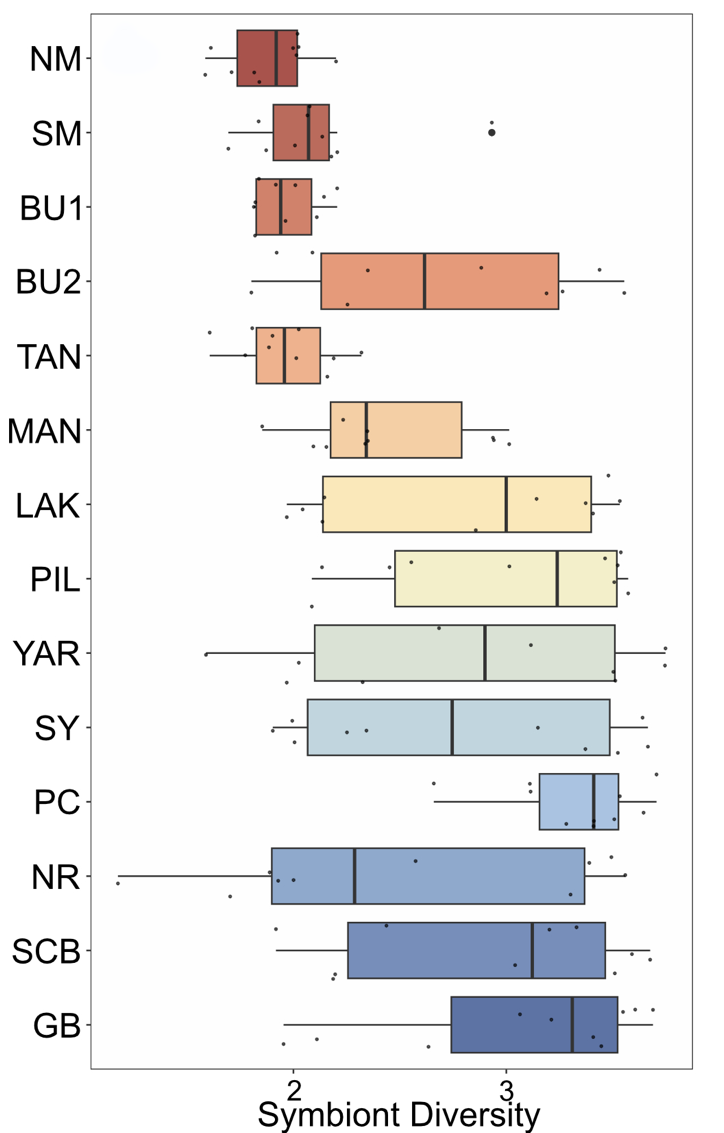


Supplementary Figure 3: Boxplots of Shannon’s diversity based on a normalised DIV count matrix of individual colonies across sites of the Ningaloo Reef Marine Park. Sites are ordered based on latitude, with warm colours denoting northern sample sites and cooler colours for southern sites.


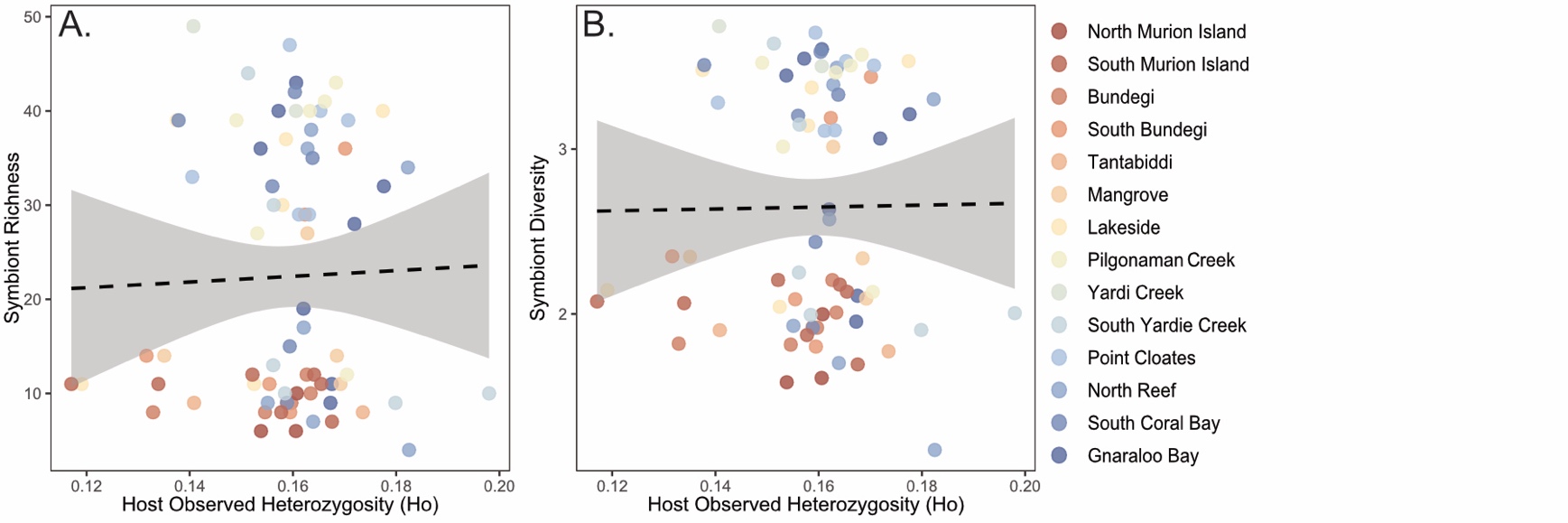


Supplementary Figure 4: Scatterplot showing the relationship between observed heterozygosity (*H*o) in the host coral and its corresponding symbiont DIV diversity (A. Richness, B. Shannon’s Diversity). Each point is an individual colony coloured by site with warm colours indicating northern sites and cool colours indicating southern sites. The black dotted line represents the linear relationship between the two.
